# Testing for deficient mismatch repair and microsatellite instability: A focused update

**DOI:** 10.1007/s00292-023-01208-2

**Published:** 2023-10-24

**Authors:** Josef Rüschoff, Hans-Ulrich Schildhaus, Jan Hendrik Rüschoff, Korinna Jöhrens, Tina Bocker Edmonston, Wolfgang Dietmaier, Hendrik Bläker, Gustavo Baretton, David Horst, Manfred Dietel, Arndt Hartmann, Frederick Klauschen, Sabine Merkelbach-Bruse, Albrecht Stenzinger, Sandra Schöniger, Markus Tiemann, Wilko Weichert, Reinhard Büttner

**Affiliations:** 1 Discovery Life Sciences Biomarker GmbH and North Hesse Pathology, Germaniastr. 7, 34119 Kassel, Germany; 2 Institute of Pathology and Molecular Pathology, Zürich University Hospital, Schmelzbergstrasse 12, 8091 Zürich, Switzerland; 3 Institute of Pathology, Carl Gustav Carus University Hospital, Fetscherstr. 74, 01307 Dresden, Germany; 4 Department of Pathology, Cooper University Health Care, 401 Haddon Ave, 08103 Camden, NJ USA; 5 Institute of Pathology/Center for Molecular Pathology Diagnosis, University of Regensburg, Franz-Josef-Strauss-Allee 11, 93053 Regensburg, Germany; 6 Institute for Pathology, Leipzig University Hospital, Leipzig, Germany; 7 Institute of Pathology, Charité University Hospital, Central Campus, Charitéplatz 1, 10117 Berlin, Germany; 8 Pathological Institute, University of Erlangen-Nuremberg, Krankenhausstr. 8–10, 91054 Erlangen, Germany; 9 Pathological Institute, Ludwig Maximilian University of Munich, Thalkirchner Str. 36, 80337 Munich, Germany; 10 Institute of Pathology, Cologne University Hospital, Kerpener Str. 62, 50937 Cologne, Germany; 11 Pathological Institute, Heidelberg University Hospital, Im Neuenheimer Feld 224, 69120 Heidelberg, Germany; 12 Hamburg Institute of Hematopathology, Fangdieckstr. 75a, 22547 Hamburg, Germany; 13 Institute of Pathology, Technical University of Munich, Trogerstr. 18, 81675 Munich, Germany

**Keywords:** Hereditary nonpolyposis colorectal neoplasms, High-throughput nucleotide sequencing, MMR immunohistochemistry, Immune checkpoint inhibitors, Lynch syndrome, Hereditäre nonpolypöse kolorektale Neoplasien, Hochdurchsatz-Nukleotidsequenzierung, MMR Immunhistochemie, Immuncheckpoint-Inhibitoren, Lynch-Syndrom

## Abstract

Testing to detect mismatch repair deficiency (dMMR) and high-grade microsatellite instability (MSI-H) has become an integral part of the routine diagnostic workup for colorectal cancer (CRC). While MSI was initially considered to be a possible indicator of a hereditary disposition to cancer (Lynch syndrome, LS), today the prediction of the therapy response to immune checkpoint inhibitors (ICI) is in the foreground. Corresponding recommendations and testing algorithms are available for use in primary diagnosis (reviewed in: Rüschoff et al. 2021).

Given the increasing importance for routine use and the expanding indication spectrum of ICI therapies for non-CRCs, such as endometrial, small intestinal, gastric, and biliary tract cancers, an updated review of dMMR/MSI testing is presented. The focus is on the challenges in the assessment of immunohistochemical stains and the value of PCR-based procedures, considering the expanded ICI indication spectrum. A practice-oriented flowchart for everyday diagnostic decision-making is provided that considers new data on the frequency and type of discordances between MMR-IHC and MSI-PCR findings, and the possible role of Next Generation Sequencing in clarifying them. Reference is made to the significance of systematic quality assurance measures (e.g., QuIP MSI portal and multicenter proficiency testing), including regular continued training and education.

**Supplementary Information:**

The online version of this article (10.1007/s00292-023-01208-2) contains Tab. S1.

## Abstract (de)

Der Nachweis der Mismatch-Reparatur-Defizienz (dMMR) mit konsekutiver hochgradiger Mikrosatelliteninstabilität (MSI-H) ist inzwischen fester Bestandteil der Diagnostik des kolorektalen Karzinoms (KRK). Galt MSI anfänglich als möglicher Indikator einer erblichen Krebsdisposition (Lynch-Syndrom, LS) steht heute die Vorhersage des Therapieansprechens auf Immuncheckpoint-Inhibitoren (ICI) im Vordergrund. Entsprechende Empfehlungen und Testalgorithmen liegen für den Einsatz in der Primärdiagnostik vor (Übersicht in: Rüschoff et al. 2021).

Aufgrund des damit verbundenen routinemäßigen Einsatzes und des sich erweiternden Indikationsspektrums von ICI-Therapien für Nicht-KRK wie Endometrium‑, Dünndarm‑, Magen- und Gallenwegskarzinome wird eine aktualisierte Übersicht zur dMMR/MSI-Testung vorgelegt. Fokus sind die Herausforderungen bei der Beurteilung immunhistochemischer Färbungen und die Wertigkeit PCR-basierter Verfahren unter Berücksichtigung des erweiterten ICI-Indikationsspektrums. Anhand neuer Daten zur Häufigkeit und Art von Diskordanzen zwischen dMMR- und MSI-Befund und der möglichen Rolle von Next Generation Sequencing zu deren Aufklärung wird ein praxisorientiertes Diagramm zur Entscheidungsfindung im diagnostischen Alltag vorgestellt. Wir weisen zudem auf die Bedeutung systematischer Qualitätssicherungsmaßnahmen (z. B. QuIP MSI-Portal und Ringversuche) einschließlich einer regelmäßigen Fortbildung hin.

The loss of a cell’s ability to repair replication errors (“mismatches”) in single repetitive (microsatellite) DNA sections is primarily caused by the biallelic inactivation of the DNA mismatch repair proteins MLH1, MSH2, MSH6, and PMS2. In most cases (70–80%), MLH1 is affected by hypermethylation of its cytidine-rich promoter regions due to increasing patient age (acquired form). Alternatively, pathogenic MMR gene mutations occur that are mainly inherited via the germline (hereditary/constitutional form), and are rarely somatically acquired (review in [25]).

Testing for deficient mismatch repair (dMMR) with consecutive high-grade microsatellite instability (MSI-H) used to be primarily recommended for patients with conspicuous family histories suggestive of a possible hereditary tumor disposition syndrome such as Lynch syndrome (LS) and related syndromes, especially in colorectal cancer (CRC) and endometrial cancer (EC). With the evidence that tumors of the dMMR/MSI‑H type demonstrate a high response rate to immune checkpoint inhibitors (ICI; [14]), the recommendation for universal testing of all CRC and EC already at the stage of the primary diagnosis has recently made its way into the corresponding therapy guidelines on a national and international level ([2], overview in [22]). In 2021, the European Medicines Agency (EMA) approved two PD‑1 targeted ICIs with pembrolizumab as first line for metastasized CRC [1], and dostarlimab as second line for recurrence or therapy failure for EC [21]. In early 2022, the indication for pembrolizumab was expanded to include unresectable or metastasized endometrial, gastric, small-intestinal or biliary cancers with MSI‑H or dMMR status [19]. The question is therefore to what extent the many years’ experience with dMMR and MSI testing for CRC (overview: [5]) can be extrapolated to the new indications, and which differences, if any, should be taken into consideration.

Immunohistochemical testing for dMMR has recently become part of the standard repertoire of any pathology laboratory, not least because of the availability of the method. This has also become increasingly true for MSI testing by polymerase chain reaction (PCR) due to simplified technology platforms [24, 30]. Several recent guidelines recommend both MMR-immunohistochemistry (IHC) and MSI-PCR as the preferred test with the European Society for Medical Oncology guidelines suggesting to use IHC first [16, 34]. Due to the now significantly expanded indication spectrum of ICI therapies, experiences with MMR-IHC and MSI-PCR testing originally coming from CRC are herein critically reviewed and reassessed.

## Significance of MMR-IHC and MSI-PCR in different organ systems

The current American Society of Clinical Oncology (ASCO)/College of American Pathologists (CAP) guideline [4] makes six recommendations, four of which address the differences in the performance of the testing methods depending on the primary tumor. Both the MMR-IHC and MSI-PCR are of equal value in the case of CRC, and next-generation sequencing (NGS) can be used if it has been validated against either of these methods (recommendation 1). In adenocarcinoma of the esophagogastric junction and the small intestine, MMR-IHC and MSI-PCR are superior to NGS (recommendation 2). Immunohistochemistry is preferred over both MSI-PCR and NGS for endometrial cancer (recommendation 3). For all other tumor entities, there were insufficient data available at the time of the literature research for the guidelines (up to February 2020). Therefore, IHC should be performed preferably until more evidence becomes available (recommendation 4).

A French group recently published their experience with a total of 3800 tumors, each tested in parallel by MMR-IHC and MSI-PCR over 10 years [10], and presented a practical approach: 15.4% (*n* = 585) of cases were diagnosed as dMMR and/or MSI‑H. Possible constellations of findings from the MMR-IHC and MSI-PCR analysis were divided into *classic findings* (84.7%, *n* = 496) with MSI‑H and loss of the respective heterodimerization proteins MLH1/PMS2 or MSH2/MSH6, and into other deviating *unusual findings* (15.2%, *n* = 89). The latter were subdivided into four groups with different constellations:

1. Isolated loss of PMS2 or MSH6
2. Loss of both heterodimer partners determined by IHC, but no MSI‑H by PCR
3. Retained MMR protein expression but MSI-H/MSI‑L determined by PCR
4. Complex immunohistochemical findings with, for example, focal (subclonal) loss of expression of an MMR protein, loss of multiple MMR proteins, or loss of MSH2 together with PMS2

**Fig. 1:**
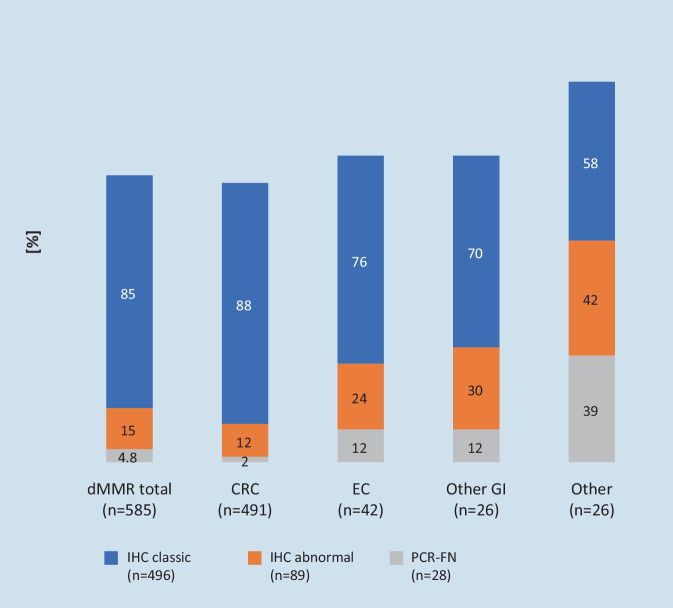
Comparison between deficient mismatch repair (*dMMR*) by immunohistochemistry (*IHC*) and microsatellite instability (MSI) status by polymerase chain reaction (*PCR*) analysis in 3800 cancers (according to Jaffrelot et al. [10], more study data: Tab. S1, online). *IHC classic:* Concordant findings between dMMR (complete loss of MLH1/PMS2 or MSH2/MSH6) and MSI-PCR (evidence of MSI-H). *IHC abnormal:* Unusual findings, e.g., isolated loss of MMR protein or contradictory findings between MMR-IHC and MSI-PCR analysis. *PCR-FN (false negative):* MSS/MSI‑L PCR finding despite classic dMMR determined by IHC. [values in columns: % of the respective tumor group] *CRC* colorectal cancer, *EC* endometrial cancer, other *GI* non-colorectal gastrointestinal tumors (9 × stomach, 5 × small intestine, 4 × duodenum, 3 × bile duct, 2 × pancreas, 1 × hepatocellular); other: 12 × sebaceous skin tumors, 6 × ovarian and 4 × urothelial cancer, and 1 × each of glioblastoma, sarcoma, melanoma, and neuro-endocrine tumor

It was shown that these unusual constellations of findings are more common in non-colorectal neoplasias and are associated with a higher probability of a false-negative PCR result **(**Fig. 1**)**.

Remarkably, cases with an unusual constellation of findings had underlying LS twice as frequently as cases with classic findings (42.7% vs. 21.4%). The majority of the 26 tumors with an unusual constellation of findings analyzed by NGS (FoundationOne® Test, Roche, Basel, Switzerland) were MSI‑H (85%) and demonstrated an increased tumor mutational burden (TMB; 21 × TMB-high, 4 × TMB-intermediate). The authors conclude that tumors with an unusual constellation of findings by MMR-IHC should not be excluded a priori from ICI therapy, and that ultimately dMMR analysis only misses a few patients potentially suitable for this therapy (< 1%).

Our previous recommendation of a step-by-step MMR/MSI diagnosis [22], starting with two MMR antibodies (PMS2, MSH6), is updated below, taking new data and recommendations into account [4, 10, 20, 31]. The focus will be on the challenges of MMR/MSI testing in everyday diagnostics, resulting from the expanded ICI therapy indication spectrum, culminating in the proposal of an optimized testing algorithm.

## Classic dMMR findings

The typical case of dMMR is characterized by a complete loss of the immunostaining for one of the two MMR protein heterodimers (MLH1/PMS2 or MSH2/MSH6) and represents the largest group by far in the French data set [10] accounting for approx. 85% of the MSI-H/dMMR findings. This finding correlates strongly with an MSI‑H status (96.9%, 496/512). The concordance between dMMR and MSI‑H was largest for CRC at 98.8% (485/491), followed by non-colorectal GI (92.9%) and endometrial cancers (91.4%). In other tumor entities the percentage of concordant dMMR and MSI‑H findings was only 79%. Accordingly, outside the colon, PCR is less sensitive and a stepwise approach starting with MMR-IHC is recommended in practice ([4, 31], review in: [22]).

## Unusual dMMR findings

All findings that deviate from the previously described classic IHC findings with complete loss of the MMR protein binding partners will be discussed here.

### Isolated loss of PMS2 or MSH6

The majority of all non-typical findings consisted of isolated loss of PMS2 or MSH6 without loss of their respective heterodimerization partners MLH1 or MSH2 (53/89). These have a prevalence of approx. 8% in dMMR CRC, 10% in EC, and 19% each in the remaining GI and other tumors. In total, 81.1% (43/53) of these cases turned out to be MSI‑H by MSI-PCR. Subsequent germline genetic testing was performed in 36 cases. In almost half of these patients a genetic background was found (45.3%): ten cases of PMS2 and 12 cases of MSH6-associated LS, and one case each of POLE-associated and constitutional MMR deficiency (CMMRD). Remarkably, 20% (5/24) of the patients with proven hereditary tumor syndrome and isolated loss of PMS2 or MSH6 were microsatellite stable (MSS) by PCR.

#### Molecular background.

Isolated loss of PMS2 or MSH6 is typically due to germline mutations of the respective gene, and is therefore associated with LS [20].

It is known that germline mutations of *MSH6* and *PMS2* demonstrate significantly lower penetrance as compared to *MLH1* and *MSH2* and confer a lower lifetime risk of cancer. Germline mutations in the *MSH6* gene in women increase the risk specifically for EC [6, 20, 32].

Accordingly, there is a trend toward a more subtle manifestation of MSI in tumors with isolated loss of MSH6 or PMS2 with only minor and discrete shifts by MSI-PCR that can easily be overlooked. In the study by Stelloo et al. [29] with 696 cases of EC, only half of the cases with isolated loss of MSH6 (*n* = 10) demonstrated an MSI‑H phenotype (Promega® System, Promega, Fitchburg, WI, USA). In another comparison study of EC, IHC proved to be superior to PCR methods including NGS. The tumor cell percentage turned out to be particularly critical for PCR techniques. It should be at least 40% in the Idylla® System (Biocartis NV, Mechelen, Belgium) instead of 20% (for CRC; [27]).

#### Recommendation.

**Fig. 2:**
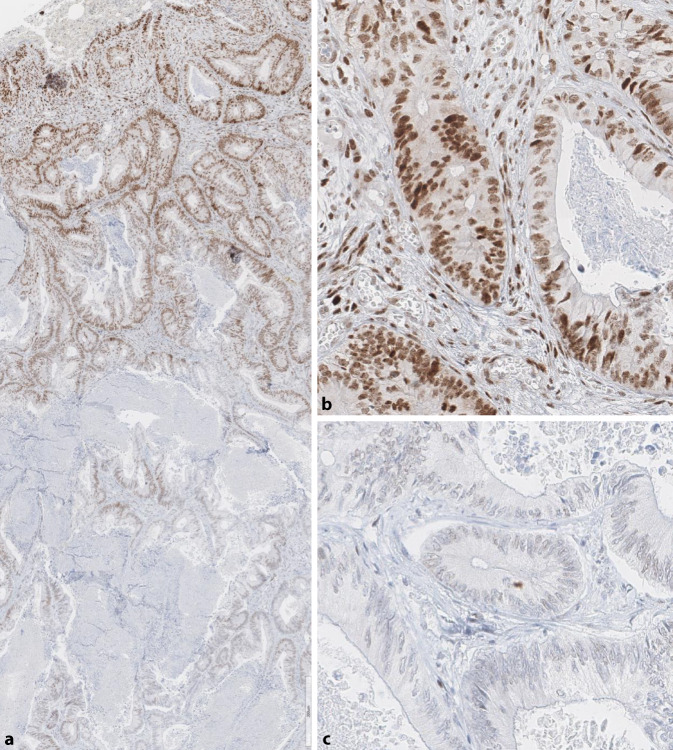
Staining gradient. Resection specimen of (**a**) colorectal cancer with decreasing staining intensity from luminal (top) to deep (bottom) areas. Only areas with proper nuclear staining of the internal controls (e.g., stroma) should be evaluated (**b**). Decreasing staining intensity in tumor and stroma indicates a fixation-related artifact; areas like the one in (**c**) must be excluded from the assessment (**a**–**c**, MLH1 antibody)

First of all, a misinterpretation of the immunohistochemical finding should be ruled out in cases of isolated loss of PMS2, e.g., due to a staining gradient and, in particular, a punctate staining pattern for MLH1 that should be interpreted as loss of the protein (Figs. 2 and 4). To further rule out MLH1 involvement, additional *BRAF* (for CRC) and/or MLH1 promoter methylation analysis for other tumor entities should be considered if necessary [20, 33]. Isolated loss of MSH6 has also been described for rectal cancer after chemoradiation [3, 8], although this is typically not associated with MSI‑H. However, the pre-treated rectal carcinoma in a patient with MSH6-related LS of the French collective was MSI‑H [10].

In principle, in cases with isolated loss of PMS2 or MSH6 confirmation of dMMR by PCR is recommended. However, a finding of MSS or MSI‑L does not rule out an underlying germline mutation. The patients should also be examined carefully for a personal and family history of cancer and should be referred to medical genetics if clinically indicated. If the PCR result is also positive (MSI-H), suitability for ICI therapy can be assumed. Most likely this is also true for patients with an MMR-deficiency syndrome and evidence of germline mutation in an MMR gene. However, there is no definitive evidence that ICI therapy will be effective in cases of isolated loss of one MMR protein and lack of MSI‑H. This constellation of findings may be a cause of therapy resistance [31]. In the cohort reported by Jaffrelot et al. [10], this affected 18.8% (10/53) of cases. An NGS analysis could be considered in which the MMR gene mutation status can be determined at the same time as the MSI and TMB status. Three of the ten patients in the study by Jaffrelot were tested further. One patient had a germline mutation in *PMS2*, one in *MSH6*, and one in *POLE* with secondary (somatic) loss of MSH6. The latter could be interpreted as eligible for ICI therapy [17]. Another case with isolated loss of PMS2 expression demonstrated a constitutional biallelic *PMS2* germline mutation (CMMRD). Cases of CMMRD are notable for the loss of MMR protein expression both in tumor and normal tissue [13] and can be missed by PCR [30]. However, these patients are also suitable for ICI therapy. In any case, this finding needs to be described in the pathology report [10].

### Discordance between MMR-IHC and MSI-PCR

Contradictory findings between IHC and PCR are described as discordant in the literature when either the MSI-PCR displays a stable phenotype (MSS/MSI-L) despite the loss of expression of an MMR protein on IHC, or there is no loss of MMR proteins on IHC despite MSI‑H findings by PCR.

The combination of dMMR determined by IHC without evidence of MSI‑H by PCR was least common in CRC at 1.2% (6/491), was more frequently observed in non-colorectal GI tumors at 7.1% and EC at 8.6% (4/46), but was most frequent in the other tumor entities at 21% (4/19; [10]). This finding was also confirmed by data from a recent analysis of four patients with LS (two patients with *MSH2* and two with *MSH6* germline mutation) with multiple tumors and/or metastases [15]. Immunohistochemistry revealed the classic dMMR finding with loss of MSH2/MSH6 in *MSH2* mutation carriers and isolated loss of MSH6 in *MSH6* mutation carriers throughout all respective primary cancers (2 × colon, 2 × rectum) and in the seven other tumors (clear cell type EC with metastasis, urothelial cancer, adrenal cancer with metastasis, 2 × sarcoma). The MSI analysis by PCR using the Bethesda and Promega® mononucleotide panel revealed an MSI‑H status in all four CRCs but in only one of the other seven tumors, which corresponds to a discordance rate of 86% in the extra-colonic tumors for these patients with LS.

#### Recommendation.

**Fig. 3:**
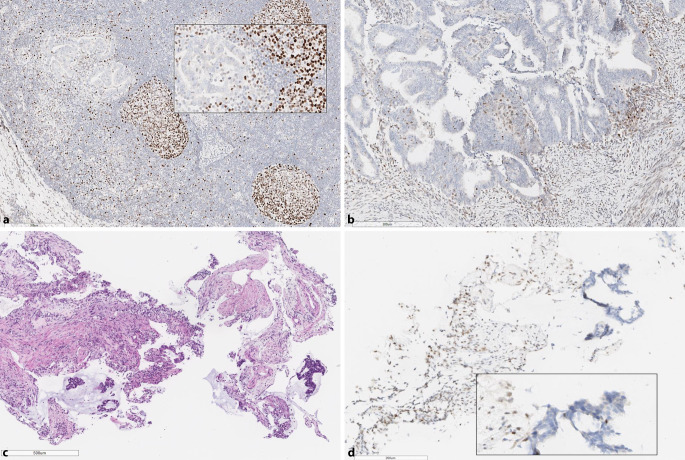
Discordance between mismatch repair–immunohistochemistry (MMR-IHC) and microsatellite instability–PCR (MSI-PCR)*: *deficient MMR (dMMR)/microsatellite stability (MSS). Group 2, according to Jaffrelot et al. [10]. **a** Lymph node metastasis of EC with isolated loss of MSH6 in cancer cells, positive reaction in follicle centers (see *inset*); negative finding (MSS) using the Idylla MSI test (overall tumor cell percentage in the lymph node approx. 20%). **b** Confirmation of isolated loss of MSH6 expression determined by IHC in the primary tumor with evidence of MSI‑H by PCR (2 out of 5 unstable loci with Idylla MSI test in a specimen with > 50% tumor cell percentage). **c** Mucinous colorectal cancer with small clusters and isolated glands of adenocarcinoma and (**d**) immunohistochemical “classic” loss of MSH2/MSH6 (MSH6, not shown) breakdown, but negative PCR findings (MSS). Evidence of MSI‑H only after targeted microdissection of tumor epithelium

In the case of dMMR determined by IHC and MSS by PCR, sample-related aspects, such as tumor cell content in the PCR sample, should be inspected first (Fig. 3). In addition, tumor biological aspects should be considered as well. The degree of instability over the course of the tumor progression increases [12] and can lead to false-negative PCR findings in early pT1 tumors. As discussed previously, depending on which MMR protein is deficient, MSI can be more or less pronounced with less prominent instability by MSI-PCR in tumors of patients harboring mutations in PMS2 and MSH6. Less prominent instability might also be missed when matching normal tissue is not analyzed by PCR (e.g., when using Idylla® system; [27].) In some cases harboring secondary mutations in the *MSH3* gene, mononucleotide repeats are less affected by instability than the longer dinucleotide and trinucleotide repeats, meaning that this MSI phenotype can be missed by test procedures that focus on mononucleotide repeats (Promega, Idylla); the Bethesda panel should be added here to increase sensitivity [5].

Given the fact that immunohistochemistry is often used as the initial screening method, cases with retained MMR protein expression but unequivocal MSI‑H by PCR (pMMR and MSI-H) are of particular concern.

However, only three cases of the 585 with evidence of pMMR and/or MSI‑H (0.5%) fulfilled this kind of discordance in the large French cohort, all of which were CRC. Two patients had LS with germline mutations, one in *MSH2* and one in *PMS2* gene. In the third patient NGS discovered a somatic double mutation in the *MLH1* and *PMS2* gene. An early study by Shia et al. reported the frequency of discordance as approx. 6% of CRC cases [9]. However, in that study, the abnormal IHC findings were included in the group with retained MMR protein expression. In a more recent study by this group [31], any deviation from the complete classic expression (pMMR) is now interpreted as abnormal, which explains the significantly lower discordance rate compared with the French data [10].

#### Recommendation.

**Fig. 4:**
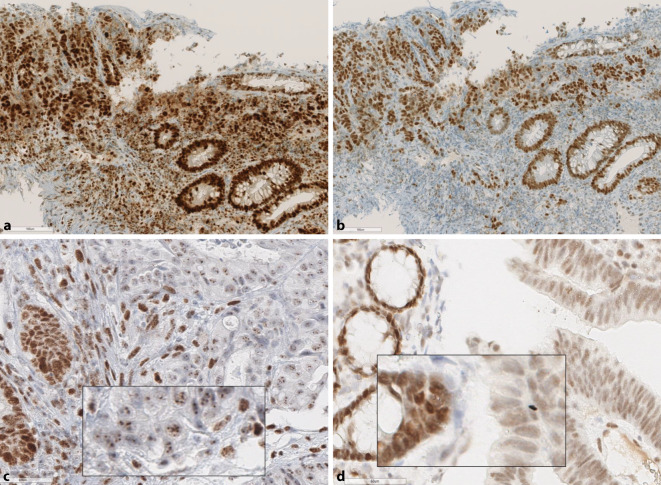
Discordance between mismatch repair–immunohistochemistry (MMR-IHC) and microsatellite instability–PCR (MSI-PCR). Group 3 according to Jaffrelot et al. [10]. **a**, **b** Biopsy of descending colon tumor (31-year-old-man), pMMR on immunohistochemistry. Strong immune reaction for PMS2 (**a**) and MSH6 (**b**) (also MLH1, MSH2, not shown). MSI-PCR performed due to the young age showed MSI‑H (Bethesda panel 3/5 microsatellite loci, Promega 3/3 microsatellite loci). On NGS analysis, a pathogenic truncating mutation close to the C‑terminus of the MSH6 gene affecting the MMR protein from amino acid 1321 on. In total MSH6 comprises 1360 amino acids and commercial antibodies typically bind to amino acid 225–450 upstream of the truncation, which most likely explains why MSH6 expression is retained (“false-positive” MMR-IHC) as those amino acids were still present. Pitfall: Incorrect interpretation of MMR-IHC as pMMR. **c** Punctate staining reactions using the MLH1 antibody (CRC shown), must not be interpreted as retained expression of MLH1 protein and is always associated with loss of PMS2 [35]. **d** Weak MSH2 staining in a CRC with complete loss of MSH6 (not shown) should not be falsely interpreted as retained MSH2 protein (intact) as the staining of the adjacent benign crypt epithelium is significantly stronger. This finding should be reported as “abnormal” according to the current recommendation [31]. The primary mutation is assumed to be in the MSH2 gene and there is no isolated loss of MSH6

To minimize the risk of false-negative MMR-IHC finding, the Bethesda criteria—especially the patient’s age—should always be considered in every case with completely maintained MMR protein expression. In younger patients (< 60 years), additional MSI-PCR analysis and human genetics consultation and/or NGS analysis to clarify the MMR gene mutation status, if necessary, is recommended (Fig. 4).

### Complex findings on IHC

**Fig. 5:**
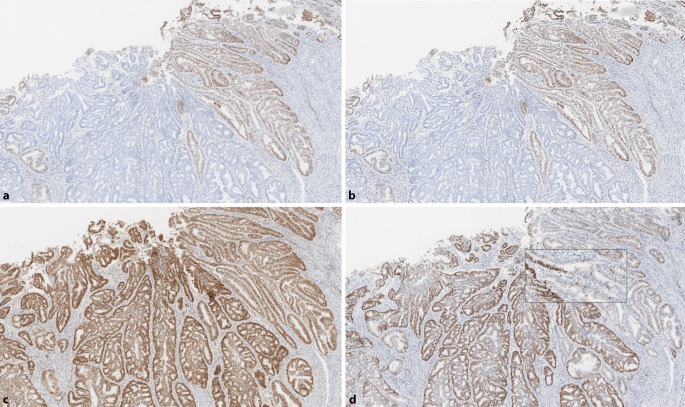
Immunohistochemical complex findings (group 4 according to Jaffrelot et al. [10]). Uterus with well-differentiated endometrial cancer and subclonal loss of MLH1 (**a**) and PMS2 (**b**) in the same tumor area and focal loss of MSH6 in different areas (**d**) while MSH2 is retained throughout the tumor (**c**). Explanation: Focal geographic loss of the MLH1/PMS2 heterodimer (affecting approx. 60% of the tumor). MSI-PCR is positive for MSI‑H and MLH1 promotor methylation is detected as the cause for the subclonal loss of MLH1/PMS2. Therefore, there is no further germline genetics work-up (see algorithm Fig. 6). Due to a secondary frame shift mutation in an intragenic C_8_ microsatellite in the *MSH6* gene, a focal loss of MSH6 protein occurs in parts of the tumor (clearly demarcated unlike the staining gradients due to fixation variations, see *inset*). This cancer was classified as dMMR and is potentially suitable for ICI therapy in the event of metastasis or relapse (recommendation: re-biopsy and re-testing)

Cases where more than just the two typical MMR protein partners forming the heterodimer are lost (in the French study: three cases with loss of three MMR proteins and four cases with loss of all four MMR proteins) represent a challenge in daily diagnostic routine. Another challenge is faint nuclear staining, which has to be interpreted with consideration of the staining intensity of internal controls such as stromal, lymphoid, and normal epithelial cells. High-quality standards and well-fixed tissue samples are required (Fig. 4d). This also applies to the interpretation of subclonal loss of MMR protein expression, which is usually reported when it affects at least 10% of the tumor (Fig. 5). These findings—still designated as not pathologic and without a link to LS in the ASCO/CAP guideline [4] and the World Health Organization classification for CRC [8]—should now be interpreted as abnormal [31]. The French data show a link to LS in more than half of cases exhibiting such complex stains (53.3%). In most of these cases (13/15), an MSI‑H status could be shown, suggesting possible response to ICI therapy.

**Fig. 6:**
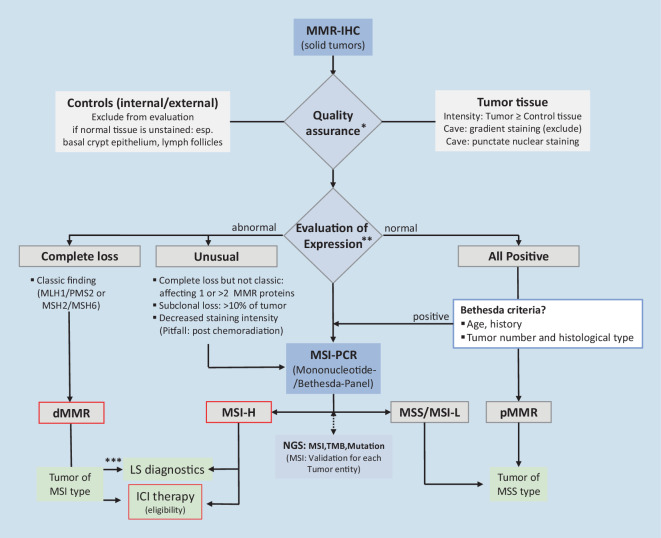
Update of step-by-step MMR and MSI status assessment of colorectal and extra-colonic tumors (pan-tumor) (according to Jaffrelot et al. [10, 31]). *) Participation in multicenter proficiency testing, e.g., QuIP (https://www.quip.eu/de), is recommended. **Two-antibody approach sufficient for classic dMMR and normal findings [22]. ***In the event of a loss of MLH1, the lack of promoter methylation (or the lack of *BRAF* mutation in case of CRC) indicates possible LS. *ICI* immune checkpoint inhibitor, *LS* Lynch syndrome, *NGS* next-generation sequencing, *MSI-PCR* microsatellite instability–polymerase chain reaction, *MSI‑H* high-grade MSI, *MSI‑L* low-grade MSI, *MSS* microsatellite stability, *MMR-IHC *mismatch repair–immunohistochemistry, *dMMR* mismatch repair deficiency, *pMMR* mismatch repair proficiency, *TMB* tumor mutational burden

#### Recommendation.

It is essential that all technical aspects of MMR-IHC are optimized and validated carefully in order to recognize such unusual complex findings determined by IHC and interpret them correctly. Areas with loss or faint staining of the internal controls must be excluded from evaluation. For tumor areas with subclonal loss of an MMR protein it is recommended to perform MSI-PCR on that area after microdissection (Fig. 3). The report should describe the findings as proposed in Table 1, including recommendation to perform further somatic mutation testing by NGS or germline testing if clinically indicated, in order to clarify the mutation status [20, 23, 31].

**Table 1:** Assessment of unusual (complex or atypical) mismatch repair (MMR)–immunohistochemical findings (according to Shia [26] and Wang et al. [31]). Staining intensity of MMR proteins in tumor (*TU*) in comparison to internal normal tissue control (*IC*): *n* negative/lost, < weakened/reduced, ~ comparable

Weak staining	TU: IC	Interpretation
MLH1/PMS2	< / ~	Normal
MLH1/PMS2	< / *n*	Both abnormal
MLH1/PMS2	~ / <	PMS2 questionable
MLH1/PMS2	*n* / <	Both abnormal
MSH2/MSH6	< or *n* / ~	MSH2 questionable; probably secondary (somatic) due to *POLE* mutation
MSH2/MSH6	< / *n*	Both abnormal
MSH2/MSH6	~ / <	MSH6 abnormal
MSH2/MSH6	*n* / <	Both abnormal
*Subclonal loss*	*Abrupt loss of expression*	*Interpretation*
MLH1/PMS2	Both	Clonal MLH1 methylation or germline mutation
MSH6 with MLH1/PMS2 dMMR	MSH6 only	Secondary mutation in coding region of *MSH6* (C8 repeat)
MLH1/PMS2, MSH2/MSH6, PMS2 alone or MSH6 alone	Both or alone	May be genetic (germline genetics work-up)
*Multiple complete loss of expression*	*Each with pos. IC*	*Interpretation*
MLH1/PMS2 and MSH6	3 proteins	Secondary mutation in coding region of *MSH6* (C8 repeat)
MLH1/PMS2 and MSH2/MSH6	4 proteins	Secondary mutation in intronic *MSH2* repeat (BAT25)

## Update on step-by-step assessment of MMR-IHC

The indication for ICI therapies being expanded to include non-CRC requires a reassessment and adaptation of the testing algorithms. New study data support the superiority of MMR-IHC compared to MSI-PCR in these indications. Supplementary molecular testing should be performed for further clarification of MMR-IHC findings that are ambiguous or unusual. MLH1 promoter methylation testing (or *BRAF* mutation testing for CRC; [33]) and NGS analysis of the tumor may be useful in clarifying the MMR gene mutation status. However, microsatellite analysis by NGS should be validated for each organ system against MMR-IHC and MSI-PCR as the reference method [10, 11, 28].

Participation in multicenter proficiency testing for quality assurance (e.g., QuIP, QuIP portal, CAP proficiency surveys) and continuing education is required to ensure high-quality performance of these important IHC biomarkers. The MMR analysis process begins with optimum fixation of the tissue to be investigated and the consideration of pre-analytical factors [18]. Optimally fixed samples are essential for detecting diagnostically relevant loss of staining, which is why biopsies should be favored over resected tissue whenever possible [7, 22]. Internal staining controls with evidence of a good-to-strong immunohistochemical reaction in the normal tissue are required for confident interpretation. If in doubt, staining of samples with only weak staining of germinal centers of lymph follicles or epithelial cells in the crypt base should be repeated or the staining protocol should be adjusted. Certain antibody clones proved to be particularly robust in multicenter ring trials (see list in [31] supplement). Ventana obtained FDA approval for MMR antibody panel.

Traditionally, only the complete loss of expression of a given MMR protein has been interpreted as dMMR [4, 8]. More recently, Wang et al. [31] suggested that only tumors with universal and complete expression of all four MMR proteins (“all present”) should be viewed as *normal* and all others as *abnormal*. This would include the typical findings of loss of the MLH1/PMS2 or MSH2/MSH6 heterodimer as well as tumors with partial, subclonal loss, or decrease in staining intensity. This approach is aligned with that of the French working group [10], which also classifies all tumors that were not completely positive or negative by MMR-IHC as “abnormal.” Hereditary syndromes (usually LS, rarely POLE or CMMRD) occurred particularly frequently (up to 50%) in this group [10]. Tumors with complete loss of an MMR protein and/or MSI‑H are potentially suitable for ICI therapy. Further studies will be needed to determine whether tumors with, for example, subclonal loss of an MMR protein without corresponding MSI‑H respond to ICI therapy.

## Practical conclusion

- DNA mismatch repair deficiency (dMMR) determined by immunohistochemistry (MMR-IHC) is closely correlated (> 98%) with the microsatellite instability (MSI) detection using PCR (MSI-PCR) in cases of colorectal cancer (CRC). Discordances between the two methods occur in approx. 5–10% of other gastrointestinal tumors and in endometrial cancer, as well as in up to 40% of other tumors.
- For cancers other than CRC, MMR-IHC shows better performance than MSI-PCR as long as stringent quality criteria (choice of antibodies, staining protocol and evaluation) are observed. Optimal fixation is essential for all methods.
- Traditionally, dMMR was defined as the complete loss of expression of an MMR protein. Today, it is recommended to view the retention of staining in the entire tumor as mismatch repair proficient (pMMR) and to classify any deviation (complete or partial loss) as dMMR (abnormal).
- In practice, IHC findings should be grouped into normal (all MMR proteins positive) and abnormal with either classic dMMR (complete loss of expression of the two corresponding proteins in the heterodimer) or abnormal findings that deviate from this. Lynch syndrome is frequently associated with the latter and can be confirmed by germline genetic testing.
- Tumors with classic dMMR and/or high-grade microsatellite instability (MSI-H) are suitable for immune checkpoint inhibitor therapy. There is uncertainty in the case of reduced/heterogeneous MMR protein expression without MSI‑H. Careful review of all findings including matching of samples, and, if necessary, further molecular investigation (e.g., next-generation sequencing) are required.

### Supplementary Information

Tab. S1: Frequency distribution of different constellations of findings in French Tumor Collective [10].

## Declarations

The supplement containing this article is not sponsored by industry.

For this article no studies with human participants or animals were performed by any of the authors. All studies mentioned were in accordance with the ethical standards indicated in each case.
