# Supplementary material for: Testing for deficient mismatch repair and microsatellite instability: A focused update
Source: Pathologie (Heidelb). 2023 Oct 24;44(Suppl 2):61–70. doi: 10.1007/s00292-023-01208-2 (PMC10713762; doi:10.1007/s00292-023-01208-2)
Supplement: Supplementary file 1 — Tab. S1: Frequency distribution of different constellations of findings in French Tumor Collective [10]. [file 292_2023_1208_MOESM1_ESM.pdf]

Tab. S1: Frequency distribution of different constellations of findings in French Tumor Collective [10]

	Classic dMMR findings	Unusual MMR-IHC findings				
	MSI-H & MLH1/PMS2 or MSH2/MSH6 loss n=496	Total n=89	isolated PMS2 or MSH6 loss Group 1, n=53	dMMR classic & MSS/MSI-L Group 2, n=16	pMMR & MSI-H/MSI-L Group 3, n=5	complex MMR protein loss Group 4, n=15
Prevalence (total)	85%	15%	59.6%	17.9%	5.6%	16.9%
Non-CRC	13.1%	32.6%	26.4%	62.5%	20.0%	26.7%
Genetic/LS	21.4%	44.9%	45.3%	37.5%	53.3%	53.3%
MLH1/PMS2	70-80%	16.8%	Group 2	11	no loss of MMR protein expression	5x all four MMR proteins
MSH2 / MSH6	20-30%	8.9%		5		3x three MMR proteins
MSH6	see unusual	38.2%	32	Group 1		2x non-heterodimer proteins
PMS2		28.1%	21			5x heterogeneous, unclear
